# Effects of voluntarily consumed sweetened alcohol and naringin on cardiac function in male and female Sprague–Dawley rats

**DOI:** 10.14814/phy2.70030

**Published:** 2024-09-08

**Authors:** Jelani Muhammad, Kennedy H. Erlwanger, Kasimu G. Ibrahim, Lebogang Mokotedi

**Affiliations:** ^1^ School of Physiology, Faculty of Health Sciences University of the Witwatersrand Johannesburg South Africa; ^2^ Department of Physiology, Faculty of Basic Medical Sciences, College of Health Sciences Federal University Birnin Kebbi Birnin Kebbi Kebbi State Nigeria; ^3^ Department of Basic Medical and Dental Sciences, Faculty of Dentistry Zarqa University Zarqa Jordan; ^4^ Department of Physiology, Faculty of Basic Medical Sciences, College of Health Sciences Usmanu Danfodiyo University Sokoto Nigeria; ^5^ Department of Physiology, School of Medicine Sefako Makgatho Health Sciences University Ga‐Rankuwa South Africa; ^6^ Integrated Molecular Physiology Research Initiative (IMPRI), School of Physiology, Faculty of Health Sciences University of the Witwatersrand Johannesburg South Africa

**Keywords:** cardiac geometry, diastolic function, naringin, sweetened alcohol

## Abstract

This study assessed the impact of sweetened alcohol and naringin on cardiac function in Sprague‐Dawley rats. Male (*n* = 40) and female (*n* = 40) rats were allocated to control, sweetened alcohol (SOH), naringin (NA), and sweetened alcohol with naringin (SOH + NA) groups. SOH and SOH + NA rats received 10% alcohol + 20% fructose in gelatine; SOH + NA and NA rats received 50 mg/kg naringin in gelatine daily for 10 weeks. Echocardiography was performed to assess left ventricular (LV) function. LV cardiomyocyte diameters and collagen area fraction were determined by H&E and picrosirius‐red staining, respectively. In males, sweetened alcohol and naringin did not affect cardiac function. Female SOH rats had increased LV end‐diastolic posterior wall (*p* = 0.04), relative wall thicknesses (*p* = 0.01), and LV cardiomyocyte diameters (*p* = 0.005) compared with control. Female SOH and SOH + NA had reduced lateral e’ and e’/a’ and increased E/e’ (*p* < 0.0001). Female SOH (*p* = 0.01) and SOH + NA (*p* = 0.04) rats had increased LV collagen area fraction compared with controls. In males, neither sweetened alcohol nor naringin affected cardiac geometry or diastolic function. In females, sweetened alcohol induced concentric remodelling, impaired LV relaxation, and elevated filling pressures. Naringin may have the potential to improve the sweetened alcohol‐induced concentric remodelling; however, it did not ameliorate diastolic dysfunction in females.

## 1 INTRODUCTION

Modern society has seen a rise in the popularity of sweetened alcoholic beverages, including flavored spirits, jello shots, and cocktails (Crews, 2015; Mosher & Johnsson, 2005; Wakabayashi et al., 2021). The sweeteners incorporated into these beverages mask the presence of alcohol, making the beverages more palatable and consequently less apparent how much alcohol is being ingested. This can increase the risk of overconsumption, particularly among adolescents who often start drinking these beverages at a young age (Binakonsky et al., 2011; Crews, 2015; Mola et al., 2023). Common sweeteners in these beverages typically include sucrose (a blend of 50% glucose and 50% fructose), high fructose corn syrup, or concentrates from fruit juice (primarily fructose) (Poppitt, 2015).

The combination of alcohol and fructose in these drinks poses a double health risk burden as individually, alcohol and fructose have been associated with negative cardiometabolic health outcomes such as obesity, dyslipidaemia, and insulin resistance (Poppitt, 2015). These modifiable risk factors may lead to the development of cardiovascular diseases such as heart failure and atherosclerosis (Liu et al., 2022; Meijers & De Boer, 2019; Tan et al., 2020). A high fructose diet may cause cardiac inflammation and fibrosis through various mechanisms including increased production of pro‐inflammatory factors, upregulation of pro‐fibrotic gene expression, macrophage infiltration, and heightened oxidative stress (Kang et al., 2015; Wang et al., 2023; Xu et al., 2022; Zhang et al., 2016). Alcohol consumption may result in increased reactive oxygen species (ROS) leading to reduced endothelial nitric oxide synthase in cardiomyocytes (Tsermpini et al., 2022; Wang et al., 2015). Therefore, the combined effects of fructose and alcohol may cause several structural changes as well as functional disturbances in the heart which may predispose individuals to cardiac dysfunction. Although fructose and alcohol individually have been extensively studied for their effect on cardiac function, their combined effects remain an area of ongoing investigation.

Managing the cardiometabolic consequences of alcohol and high fructose consumption results in a heavy socioeconomic burden for many countries globally (Rao, 2018). There is a need to explore prophylactic interventions early in life to reduce the poor health outcomes associated with alcohol and sugar consumption. Phytochemicals are increasingly being targeted as candidates for nutraceutical potential (Bruno et al., 2022).

Naringin, a flavonoid phytochemical found in citrus fruit, has recently become of interest for its possible cardioprotective effects (Akin et al., 2022; Malakul et al., 2018; Park et al., 2018; Shackebaei et al., 2023; Uryash et al., 2021). Studies have reported that naringin has antioxidant and anti‐inflammatory properties (Akin et al., 2022; Uryash et al., 2021) that may protect the myocardium by reducing inflammation as well as oxidative stress that takes place after the consumption of fructose or alcohol. Nevertheless, to the best of our knowledge, there is no literature regarding the effects of naringin on sweetened alcohol‐induced cardiac changes. Consequently, further studies are required to determine naringin's potential cardioprotective effects in the context of sweetened alcoholic beverage consumption. There is a bias towards the use of males in animal‐based studies despite the societal trends showing that both males and females consume sweetened alcoholic beverages (Wakabayashi et al., 2021). Therefore, this research aimed to investigate the effects of sweetened alcohol and naringin on cardiac function in male and female Sprague–Dawley rats.

## 2 METHODS

### 2.1 Ethical clearance and study site

The study adhered to international ethical guidelines and standards governing the care and utilization of laboratory animals, receiving approval from the Animal Research Ethics Committee of the University of Witwatersrand (AREC clearance certificate number 2022/10/02/C). The research took place at the Wits Research Animal Facility (WRAF) situated at the University of the Witwatersrand in Johannesburg, South Africa. The study adhered to the Animal Research Reporting of In Vivo Experiments (ARRIVE) guidelines both during the research process and in the compilation of the manuscript.

### 2.2 Animals and study design

A total of 80, 42‐day‐old Sprague–Dawley rats (comprising 40 males and 40 females), were sourced from the WRAF of the University of the Witwatersrand, Johannesburg. Each rat was housed in a perspex cage topped with steel mesh covers. The cages were lined with wood shavings and shredded paper for bedding and environmental enrichment. Additional environmental enrichment included wooden blocks for the rats to gnaw on and sections of wide bore pipes for the rats to hide in. Rats were provided with ad libitum access to standard pelletized rat chow (LabChef®, Johannesburg, South Africa) and tap water for the entirety of the study. To maintain a hygienic environment, cages, and bedding were changed twice a week. The room's ambient temperature was controlled at 26 ± 2°C, with appropriate ventilation and a 12‐h light/dark cycle (lights on at 07.00 h).

The experiment was designed as a randomized, interventional prospective study. The rats were subjected to a 1‐week acclimatization period wherein initial assessments, including body mass, food consumption, and blood pressure were recorded. The rats were also acclimatized with gelatine which was prepared by adding 0.5 g fructose with 6 g gelatine in 100 mL of water. It was given daily at 10 mL/100 g body weight for all the rats during the acclimatization period. Following this acclimatization period, rats were randomly assigned to one of four treatment groups, each consisting of 20 rats (10 males and 10 females):

1. Control: received plain gelatine (0.5% fructose in 6% gelatine)—fructose was added to the gelatine to improve the palatability of the preparation.
2. Sweetened alcohol (SOH): received sweetened alcohol (10% alcohol +20% fructose) in 6% gelatine.
3. Naringin (NA): received 50 mg/kg body mass (BM) naringin in gelatine (0.5% fructose in 6% gelatine).
4. Sweetened alcohol + naringin (SOH + NA): received sweetened alcohol (10% alcohol +20% fructose) + 50 mg/kg BM naringin in 6% gelatine.

The gelatine with or without alcohol and/or naringin was provided daily to the rats in glass containers at a volume of 10 mL per 100 g body mass. The use of gelatine for voluntary administration of alcohol has been previously described and reported in various studies (Dess et al., 2013; Peris et al., 2006; Ralevski et al., 2006).

**FIGURE 1:**
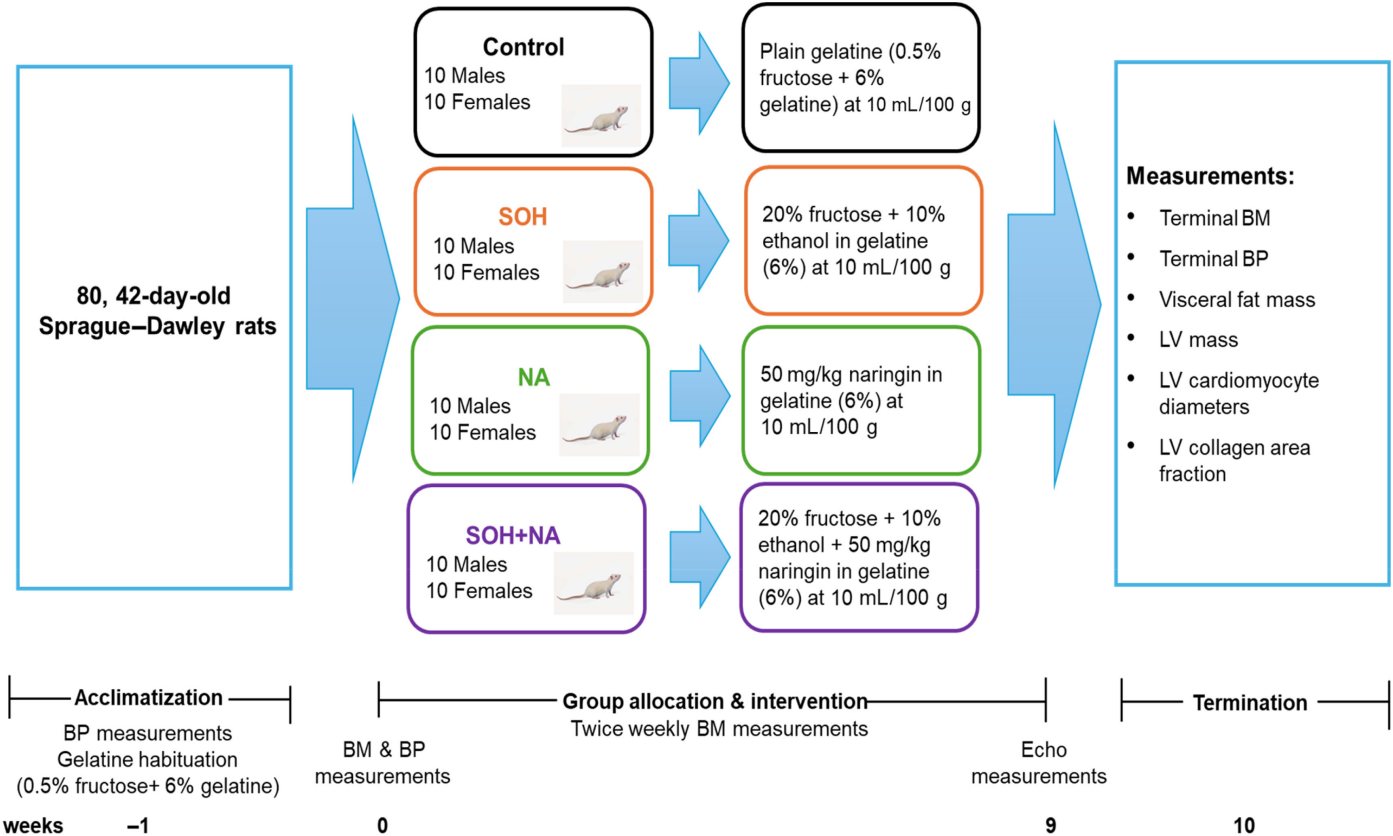
Schematic diagram of the study design. BP, blood pressure; BM, body mass; Echo, echocardiography; LV, left ventricle; NA, naringin; SOH + NA, sweetened alcohol and naringin; SOH, sweetened alcohol.

Before the commencement of the intervention, baseline blood pressure and body masses were measured. The study design is depicted in Figure 1 below.

### 2.3 Interventions used and preparation

For our study, gelatine (Sheridan's gelatine, Libstar Operations (Pty) Ltd, Dunkeld, South Africa) was used as a vehicle. Plain gelatine was prepared by mixing 6 g gelatine with 0.5 g fructose powder (Fructose, Nature's Choice, Randvaal, South Africa) and 1–2 mL Bovril (Beefy Bovril, Pioneer Foods (Pty) Ltd, South Africa) in 100 mL of hot water. Naringin was incorporated into gelatine by adding 0.05 g (to provide a dose of 50 mg/kg body mass (Adebiyi et al., 2016)) naringin (>95% HPLC, CAS No. 10236‐47‐2, Sigma‐Aldrich, China) to the plain gelatine as described above. Sweetened alcohol in gelatine was prepared by adding 20 g fructose powder (equivalent to 20% fructose (Komnenov et al., 2020)), 6 g gelatine, and 1–2 mL Bovril, with 10 mL of ethanol (equivalent to 10% ethanol (Al‐Awwadi et al., 2004)) (Ethanol 99.9%; CAS No. 64‐17‐5; Batch No. 37500; ACE chemicals, South Africa) to provide up to a maximum ethanol dose of 0.79 g/100 g body weight, in 90 mL of hot water (<60°C). This dose is equivalent to about half of a glass/unit of alcohol in humans which corresponds to chronic light alcohol consumption (Brick, 2006) and has been associated with elevated liver enzymes (Niemelä et al., 2017). Sweetened alcohol with naringin was prepared by adding 0.05 g naringin (to provide a dose of 50 mg/kg body mass) of naringin into the sweetened alcohol in gelatine preparation as described above (doses were adjusted to the rats' body mass). In each case, the mixture was thoroughly mixed using a magnetic hot plate stirrer. The solutions/mixtures were then placed into glass containers and sealed to prevent evaporation of the alcohol. All preparations were kept and allowed to set in a fridge at 2–7°C for 4–24 h before being served to the rats. All treatments (either plain gelatine, sweetened alcohol, naringin in gelatine, or sweetened alcohol with naringin) were offered to the rats once daily (at approximately 2:00 pm) at a dosage of 10 mL per 100 g body mass over 10 weeks. The gelatine preparation consumed by each rat was estimated by subtracting the remaining gelatine in milliliters (mL) from the gelatine provided each week. Relative gelatine consumption was determined by dividing the gelatine consumed by body mass and then multiplying by 100.

### 2.4 Body mass measurements

An electronic weighing balance (Snowrex EQ‐1200, Snowrex International Company, Taipei, Taiwan) was used to measure the baseline body mass. Subsequently, measurements were conducted twice weekly to assess growth progress and make necessary adjustments to the dosage of fructose, gelatine, ethanol, and naringin, thereby ensuring a consistent dosage proportionate to the body mass. The final body mass was measured at termination.

### 2.5 Noninvasive blood pressure measurements

Blood pressure was measured at baseline and termination using the tail‐cuff technique (Biopac Systems, Santa Barbara, CA, USA), as previously described (Pauline et al., 2011). Briefly, each rat (priorly habituated to the procedure) was put in a restrainer, with the rat's tail on a heating pad. After the rats' tails were heated, the blood pressure machine's cuff was placed at the base of the tail, and readings of blood pressure were recorded. Two to three readings per rat were taken and the average was determined. To prevent diurnal variation, measurements were done at midday.

### 2.6 Echocardiography measurements

Nine weeks after the commencement of the intervention, rats were anesthetized with isoflurane (1%–3% in oxygen). Anesthesia was first induced in a chamber and then maintained with a face mask. Following this, the anterior chest wall was shaved.

Echocardiography was performed with the rats in the left lateral decubitus position using a high‐resolution ultrasound probe (10 MHz) coupled to an echocardiographic machine (Affiniti CVx, Philips Healthcare, Andover, Massachusetts). LV dimensions were determined using two‐dimensional directed M‐mode echocardiography in the parasternal long‐axis view during three consecutive beats. LV end‐systolic and end‐diastolic internal diameters, interventricular septal thickness, and posterior wall thickness were measured in systole and diastole. Relative wall thickness was calculated as (2 × posterior wall thickness)/(LV internal diameter at end‐diastole) (Lang et al., 2015). LV pump and myocardial systolic function were determined by calculating LV ejection fraction using the Teichholz method and LV endocardial fractional shortening (Lang et al., 2015).

LV diastolic function was determined from the mitral valve inflow patterns using pulsed Doppler imaging. In the apical 4‐chamber view, the early (E) and late (A) diastolic inflow velocities were obtained with the sample volume placed at the mitral valve leaflet tip and expressed as E/A, a marker of relaxation. To determine diastolic function using tissue Doppler imaging (TDI), peak myocardial tissue lengthening velocities during early (e') and late (a') diastole were recorded at the lateral mitral annulus in the apical four‐chamber view. Diastolic function markers are expressed as e' (an index of myocardial relaxation), e'/a' (an index of myocardial stiffness), and E/e' (an index of LV filling pressure).

The rats were allowed to recover from the anesthetic and then returned to their cages and respective treatments for a week.

Measurements were taken in the LV since the LV plays a crucial role in ensuring blood circulates to all parts of the body. Given its thicker wall compared to the right ventricle, the LV is more often susceptible to various cardiovascular conditions, and therefore, studying the left side of the heart provides meaningful information into the overall cardiac function (Kerfoot et al., 2019). Moreover, cardiovascular diseases such as heart failure, myocardial infarction, and hypertension, predominantly affect the left side of the heart (Tsao et al., 2015). Therefore, focusing on the left side provides a better understanding of the mechanisms associated with these conditions.

### 2.7 Terminal procedures

At the end of the 10‐week intervention, rats were euthanized using a sodium pentobarbital overdose (Euthapent, Kyron laboratories, Johannesburg, South Africa) at 150 mg/kg BM injected intraperitoneally. Visceral fat (adjoining the liver, kidneys, pancreas, stomach, and small and large intestines) was carefully removed, and weights were determined using a Presica 310 M digital scale (Precision Instruments, Johannesburg, South Africa). The hearts were dissected out and their respective weights were recorded. Subsequently, the left ventricle was isolated from the other cardiac structures, and its specific weight was determined using a Presica 310 M digital scale (Precision Instruments, Johannesburg, South Africa).

### 2.8 LV cardiomyocyte diameter and total collagen content determination

LV tissue was preserved in 10% phosphate‐buffered formalin and later histologically examined. For the histological examination, the samples were processed using the Thermo Scientific's Microm STP 120 (Microm STP 120, Thermo Scientific, MA, USA) automatic tissue processor, and embedded in paraffin wax. Processed tissues were sectioned at 4 μm thick using a microtome (Leica Biosystems Instruments (Pty), Ltd, model RM 2125, Wetzlar, Germany) and placed onto glass microscope slides. The slides were allowed to air‐dry and adhere to a 20°C heating block.

#### 2.8.1 Hematoxylin and eosin stain

The Hematoxylin and Eosin (H&E) stain was used to assess LV cardiomyocyte diameter (width) at the level of the nucleus in longitudinal sections. Briefly, the samples underwent deparaffinization with xylene, rehydration through a series of graded ethanol concentrations, and staining with Harris' hematoxylin followed by alcoholic eosin solution. Subsequently, the slides were washed (dehydrated) with ethanol and xylene before being mounted with DPX mountant and covered with a coverslip. The slides were visually analyzed by a blinded evaluator using a Zeiss Axioskop 2 Plus microscope, equipped with a Zeiss AxioCam (Zeiss, Peabody, MA, USA), to analyze the LV cardiomyocyte diameter. Tissue sections viewed under a bright field were obtained using a 4× objective lens (×400 magnification). The LV cardiomyocyte diameters at the level of the nucleus in longitudinal sections were determined using the straight‐line tool on ImageJ software (ImageJ version 1.54d, Laboratory for Optical and Computational Instrumentation, University of Wisconsin) (Baudouy et al., 2017). LV cardiomyocyte diameters were measured three times per sample and an average was determined.

#### 2.8.2 Picrosirius red stain

The picrosirius red stain was used to measure the accumulation of collagen fibers to quantify the degree of fibrosis in LV samples. Briefly, sections were deparaffinized, rehydrated, and stained with a 0.1% Sirius Red solution dissolved in aqueous saturated picric acid for 60 min at room temperature. After washing in acidified water (5 mL of acetic acid into 1 L of distilled water), the slides were dehydrated and mounted with DPX mountant (Merck Life Science (Pty) Ltd, Modderfontein, South Africa). The tissue sections were visually analyzed by a blinded evaluator using a Zeiss Axioskop 2 Plus microscope equipped with a Zeiss AxioCam (Zeiss, Peabody, MA, USA). Tissue section images viewed under bright‐field and polarized light were obtained using a 10× objective lens (x100 magnification). The collagen area fraction and overall tissue area for each tissue section were determined using ImageJ software (ImageJ version 1.54d, Laboratory for Optical and Computational Instrumentation, University of Wisconsin). Collagen area fractions were determined three times per sample and an average was determined. The collagen area fraction was computed by dividing the collagen area by the overall tissue area (Baidoo et al., 2022).

### 2.9 Statistical analysis

Statistical analysis was performed using SAS version 9.4 (SAS Institute Inc. Cary, North Carolina, USA). Graphs were generated using GraphPad Prism version 9.4 (GraphPad Software Inc., San Diego, CA, USA).

The sample size was determined using the formula (Charan & Kantharia, 2013): 2 SD^2^ (1.96 + 0.842)^2^/d^2^ where SD = standard deviation; 1.96 = type 1 error of 5%; 0.842 = at 80% power; 0.842 is a value from the standard formula used to determine the sample size. The value (0.842) is associated with the Z‐score, or critical value related to achieving 80% power in a standard normal distribution. When using a standard normal distribution, a Z‐score of approximately 0.842 corresponds to 80% power (Charan & Kantharia, 2013), and d = difference between mean values.

Shapiro–Wilk tests were used for normality tests. Data were normally distributed and are expressed as means ± SEM. To determine differences in baseline and terminal body masses and blood pressure measurements, as well as weekly gelatine consumption measures, repeated measures two‐way analysis of variance (ANOVA) was used with group and sex as the main effects. If significant differences were observed, Tukey post hoc tests were employed to identify specific group and time differences. Cardiac geometry and functional differences were assessed using two‐way ANOVA with group and sex as the main effects. If significant differences were observed, Tukey post hoc tests were employed to identify specific group differences. Associations between tissue Doppler indices and the collagen area fraction were determined using Pearson's correlation. Since blood pressure contributes to the development of diastolic dysfunction, blood pressure was included as a confounder in the correlation analysis. *p* < 0.05 was considered as significant.

## 3 RESULTS

### 3.1 Morbidity and mortality

One of the male rats died from the Male SOH + NA group during the study due to iatrogenic causes (confirmed by autopsy).

### 3.2 Body mass, visceral fat mass, and blood pressure measurements

Table 1 shows the effects of sweetened alcohol and naringin on body mass and blood pressure at baseline and at termination in male and female Sprague–Dawley rats. The group and sex effects were significantly different for baseline body mass (group: *F* = 2.49, *p* = 0.02; sex: *F* = 13.27, *p* = 0.0005) and terminal body mass (group: *F* = 175.60, *p* < 0.0001; sex: *F* = 1208.68, *p* < 0.0001). Body masses were significantly higher (*p* < 0.0001) at termination compared to baseline across all groups in both male and female rats. Males were heavier (*p* < 0.0001) compared to females at baseline and termination.

**TABLE 1:** Effects of sweetened alcohol and naringin on body mass, visceral fat mass, and blood pressure at baseline and termination in male and female Sprague–Dawley rats.

	Males				Females			
	Control	SOH	NA	SOH + NA	Control	SOH	NA	SOH + NA
Baseline								
Body mass (g)	148 ± 6^**δ**^	144 ± 6^**δ**^	154 ± 6^**δ**^	156 ± 6^**δ**^	118 ± 6^**δ*α***^	122 ± 6^**δ*α***^	111 ± 6^**δ*α***^	132 ± 6^**δ*α***^
SBP (mm Hg)	116 ± 4	119 ± 2	124 ± 3	118 ± 4	119 ± 3	120 ± 3	120 ± 3	122 ± 3
DBP (mm Hg)	79 ± 2	75 ± 3	72 ± 2	76 ± 3	75 ± 3	78 ± 3	75 ± 3	79 ± 2
Termination								
Body mass (g)	463 ± 6	435 ± 6	460 ± 6	428 ± 6	258 ± 6^***α***^	271 ± 6^***α***^	257 ± 6^***α***^	268 ± 6^***α***^
Visceral fat (% body mass)	2.79 ± 0.38	2.74 ± 0.38	2.87 ± 0.41	2.87 ± 0.41	3.97 ± 0.38	6.17 ± 0.38*^# ***α***^	4.09 ± 0.38	6.64 ± 0.38*^# ***α***^
SBP (mm Hg)	117 ± 1	115 ± 3	119 ± 3	117 ± 2	119 ± 3	117 ± 2	117 ± 2	119 ± 3
DBP (mm Hg)	79 ± 2	79 ± 2	76 ± 2	78 ± 3	78 ± 2	79 ± 1	81 ± 1	77 ± 1

*Note*: Data expressed as mean ± SEM. *p < 0.05 compared to control. ^#^ *p* < 0.05 compared to NA. ^δ^ *p* < 0.0001 compared to terminal body mass. ^α^ *p* < 0.0001 compared to all male groups.

Abbreviations: DBP, diastolic blood pressure; NA, naringin; SBP, systolic blood pressure; SOH + NA, sweetened alcohol and naringin; SOH, sweetened alcohol.

The group and sex effects were significantly different for visceral fat mass indexed to body mass (group: *F* = 16.44, *p* < 0.0001; sex: *F* = 76.13, *p* < 0.0001). Female SOH and SOH + NA rats had heavier visceral fat masses indexed to body mass compared to female control and NA rats (*p* < 0.0001). Female SOH and SOH + NA rats had heavier visceral fat masses indexed to body mass compared to all male groups (*p* < 0.0001).

There were no significant differences observed in group and sex effects for baseline systolic blood pressure (group: *F* = 0.42, *p* = 0.89; sex: *F* = 0.13, *p* = 0.72) and terminal systolic blood pressure (group: *F* = 0.81, *p* = 0.58; sex: *F* = 0.11, *p* = 0.74). There were no significant differences observed in group and sex effects for baseline diastolic blood pressure (group: *F* = 1.13, *p* = 0.31; sex: *F* = 0.02, *p* = 0.92) and terminal diastolic blood pressure (group: *F* = 1.15, *p* = 0.34; sex: *F* = 0.00, *p* = 0.96).

### 3.3 Gelatine consumption

**FIGURE 2:**
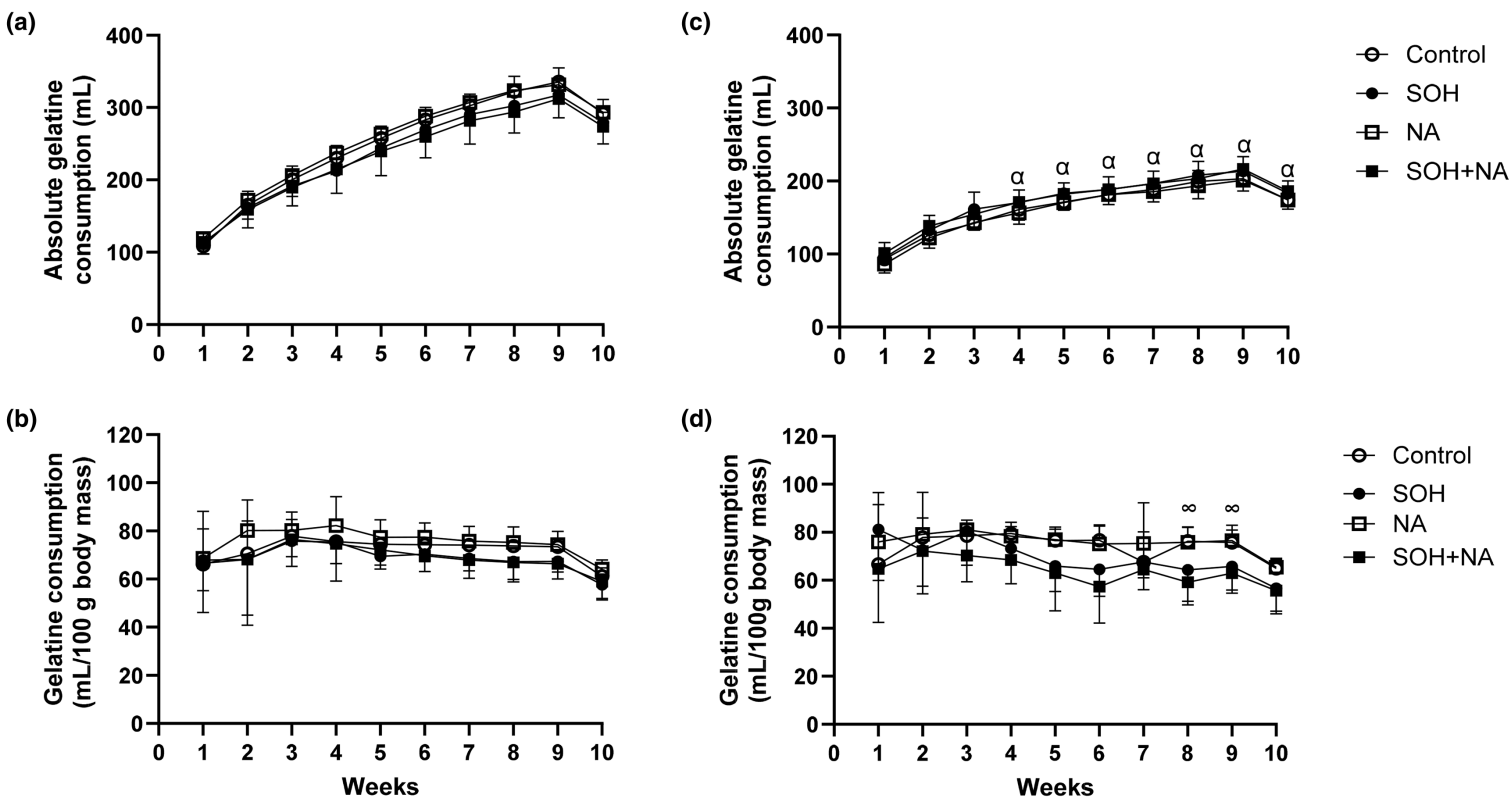
The weekly absolute (a and c) and relative (b and d) gelatine consumption in male (left panel) and female (right panel) Sprague–Dawley rats. Data presented as means ± SEM. ^∞^ *p* < 0.05 control and NA compared to SOH + NA. ^α^ *p* < 0.05 compared to all male groups. NA, naringin; SOH + NA, sweetened alcohol and naringin; SOH, sweetened alcohol.

Figure 2 shows the effects of sweetened alcohol and naringin on weekly absolute and relative gelatine consumption in male and female Sprague–Dawley rats. The group and sex effects were significantly different for absolute gelatine consumption (group: *F* = 54.81; *p* < 0.0001; sex: *F* = 365.25; *p* < 0.0001). There were no significant differences in absolute gelatine consumption between the groups in both males and females for all 10 weeks (Figure 2a,c). Male rats had higher absolute gelatine consumption compared to females from week 4 to week 10 (*p* < 0.01; Figure 2a,c).

When gelatine consumption was computed relative to body mass, the group effect was significant (*F* = 11.78; *p* < 0.0001), however, the sex effect was not (*F* = 0.48; *p* = 0.48). There were no significant differences in relative gelatine consumption between the groups in males for all 10 weeks (Figure 2b). In females, the relative gelatine consumption was higher in the control and NA groups compared to the SOH + NA group at week 8 (*p* = 0.02 and *p* = 0.03) and week 9 (*p* = 0.03 and *p* = 0.03; Figure 2d).

### 3.4 Echocardiography

#### 3.4.1 Cardiac mass, cardiac geometry, and left ventricular systolic function

Table 2 shows the effects of sweetened alcohol and naringin on cardiac mass, cardiac geometry, and left ventricular systolic function in male and female Sprague–Dawley rats. The group and sex effects were significantly different for relative heart masses (group: *F* = 21.26, *p* < 0.0001; sex: *F* = 138.72, *p* < 0.0001) and absolute heart masses (group: *F* = 6.54, *p* < 0.0001; sex: *F* = 34.03, *p* < 0.0001). The group and sex effects were significantly different for relative LV masses (group: *F* = 28.42, *p* < 0.0001; sex: *F* = 195.68, *p* < 0.0001) and absolute LV masses (group: *F* = 3.62, *p* < 0.0001; sex: *F* = 18.11, *p* < 0.0001). Male heart and LV masses were heavier (*p* < 0.0001) compared to female heart and LV masses. However, female control, NA, and SOH + NA groups had heavier (*p* < 0.01) heart mass indexed to body mass compared to male control and SOH groups. Female control, NA, and SOH + NA groups had heavier (*p* < 0.01) LV mass indexed to body mass compared to the male control group.

**TABLE 2:** Effects of sweetened alcohol and naringin on cardiac masses and geometry, and left ventricular systolic function in male and female Sprague–Dawley rats.

	Males				Females			
	Control	SOH	NA	SOH + NA	Control	SOH	NA	SOH + NA
Cardiac masses and geometry								
Heart mass (g)	1.63 ± 0.06	1.55 ± 0.06	1.80 ± 0.06	1.64 ± 0.06	1.14 ± 0.02^**α**^	1.19 ± 0.10^**α**^	1.11 ± 0.04^**α**^	1.16 ± 0.05^**α**^
Heart mass/body mass x 10^3^	3.52 ± 0.12^∞^	3.57 ± 0.13	3.52 ± 0.20^∞^	3.90 ± 0.12	4.41 ± 0.09	4.03 ± 0.09	4.32 ± 0.19	4.33 ± 0.12
LV mass (g)	0.68 ± 0.03	0.72 ± 0.03	0.72 ± 0.02	0.73 ± 0.02	0.47 ± 0.02^**α**^	0.48 ± 0.02^**α**^	0.49 ± 0.03^**α**^	0.50 ± 0.02^**α**^
LV mass/body mass × 10^3^	1.48 ± 0.07^∞^	1.67 ± 0.07	1.59 ± 0.05	1.73 ± 0.04	1.83 ± 0.06	1.70 ± 0.06	1.89 ± 0.11	1.82 ± 0.08
LV end‐diastolic diameter (mm)	7.50 ± 0.16	7.33 ± 0.16	7.46 ± 0.17	7.41 ± 0.16	5.46 ± 0.17^**α**^	5.29 ± 0.17^**α**^	5.43 ± 0.16^**α**^	5.32 ± 0.16^**α**^
LV end‐diastolic posterior wall thickness (mm)	1.70 ± 0.07	1.78 ± 0.07	1.66 ± 0.07	1.72 ± 0.08	1.27 ± 0.08^**α**^	1.62 ± 0.08*^#^	1.31 ± 0.07^**α**^	1.56 ± 0.07
LV end‐systolic diameter (mm)	3.86 ± 0.16	3.87 ± 0.16	3.88 ± 0.16	3.89 ± 0.17	3.11 ± 0.17^**α**^	3.19 ± 0.17	3.09 ± 0.16^**α**^	3.22 ± 0.16
LV end‐systolic posterior wall thickness (mm)	3.12 ± 0.11	3.11 ± 0.11	3.06 ± 0.11	3.12 ± 0.11	2.51 ± 0.11^**α**^	2.48 ± 0.11^**α**^	2.51 ± 0.11^**α**^	2.49 ± 0.11^**α**^
Relative wall thickness	0.46 ± 0.02^β^	0.49 ± 0.02	0.45 ± 0.02^β^	0.47 ± 0.03	0.47 ± 0.03	0.62 ± 0.03*^#**α**^	0.49 ± 0.02	0.57 ± 0.02
Systolic function								
LV stroke volume (mL)	0.53 ± 0.03	0.50 ± 0.03	0.53 ± 0.03	0.51 ± 0.03	0.41 ± 0.03	0.40 ± 0.03	0.40 ± 0.03	0.40 ± 0.03
Ejection fraction (%)	80.31 ± 2.19	78.27 ± 2.19	78.74 ± 2.19	78.21 ± 2.31	81.99 ± 2.31	79.74 ± 2.31	82.04 ± 2.19	78.76 ± 2.19
Fractional shortening (%)	48.31 ± 2.51	46.94 ± 2.51	47.93 ± 2.51	47.29 ± 2.65	42.87 ± 2.65	39.74 ± 2.65	41.88 ± 2.51	41.02 ± 2.51

*Note*: Data presented as mean ± SEM. *p < 0.05 compared to control. ^**#**^ *p* < 0.05 compared to NA. ^α^ *p* < 0.05 compared to all male groups. ^β^ *p* < 0.05 compared to female SOH + NA group. ^∞^ *p* < 0.05 compared to female control, NA and SOH + NA.

Abbreviations: LV, left ventricle; NA, naringin; SOH + NA, sweetened alcohol and naringin; SOH, sweetened alcohol.

The group and sex effects were significantly different for LV end‐diastolic diameter (group: *F* = 42.75, *p* < 0.0001; sex: *F* = 297.96, *p* < 0.0001), LV end‐diastolic posterior wall thickness (group: *F* = 6.72, p < 0.0001; sex: *F* = 27.97, *p* < 0.0001), LV end‐systolic diameter (group: *F* = 5.41, p < 0.0001; sex: *F* = 37.47, *p* < 0.0001), LV end‐systolic posterior wall thickness (group: *F* = 8.90, *p* = 0.0001; sex: *F* = 62.04, *p* < 0.0001), and relative wall thickness (group: *F* = 5.71, *p* < 0.0001; sex: *F* = 17.17, *p* < 0.0001) in male and female rats. Female SOH rats had greater LV end‐diastolic posterior wall thickness, and relative wall thickness compared to female control rats (*p* = 0.04, and *p* = 0.01, respectively). Female SOH rats had greater relative wall thickness compared to female NA rats (*p* = 0.01). No significant differences in relative wall thickness were observed between the female control, NA, and SOH + NA groups. LV end‐diastolic diameter and LV end‐systolic posterior wall thickness were greater (*p* < 0.001) in all males compared to all female groups. Female control and NA rats had reduced (*p* < 0.01) LV end‐diastolic posterior wall thickness and LV end‐systolic diameter compared to all male groups. Female SOH rats had greater (*p* < 0.01) relative wall thickness compared to all male groups. Female SOH + NA rats had greater (*p* < 0.05) relative wall thickness compared to male control and NA groups.

The group and sex effects were significantly different for LV stroke volume (group: *F* = 4.04, *p* = 0.0009; sex: *F* = 27.56, *p* < 0.0001). There were no significant differences in group or sex effects for ejection fraction (group: *F* = 0.49, *p* = 0.84; sex: *F* = 1.19, *p* = 0.28). Although the sex effect was significantly different for fractional shortening (*F* = 11.84, *p* = 0.0010), no significant differences were observed for the group effect (*F* = 1.83, *p* = 0.11). There were no significant differences between groups in all markers of systolic function in male and female rats.

#### 3.4.2 Left ventricular diastolic function

##### Pulsed Doppler indices

Table 3 shows the effects of sweetened alcohol and naringin on pulsed Doppler indices (E and E/A) in male and female Sprague–Dawley rats. There were no significant differences observed in the group and sex effects for E velocity (group: *F* = 0.12, *p* = 0.99; sex: *F* = 0.00, *p* = 0.96) and E/A (group: *F* = 0.00, *p* = 1.00; sex: *F* = 0.01, *p* = 0.91) in male and female rats.

**TABLE 3:** Effects of sweetened alcohol and naringin on left ventricular diastolic function (pulsed Doppler indices) in male and female Sprague–Dawley rats.

	Males				Females			
	Control	SOH	NA	SOH + NA	Control	SOH	NA	SOH + NA
E (cm/s)	70.75 ± 3.13	73.20 ± 3.13	70.70 ± 3.13	70.89 ± 3.30	70.00 ± 3.30	72.06 ± 3.30	70.60 ± 3.13	72.50 ± 3.13
E/A	1.67 ± 0.13	1.65 ± 0.13	1.66 ± 0.13	1.65 ± 0.14	1.68 ± 0.14	1.66 ± 0.14	1.66 ± 0.13	1.67 ± 0.13

*Note*: Data presented as means ± SEM.

Abbreviations: A, late diastolic inflow velocities; E, early; E/A, ratio of early and late diastolic inflow velocities; LV, left ventricle; NA, naringin; SOH + NA, sweetened alcohol and naringin; SOH, sweetened alcohol.

##### Tissue Doppler indices

**FIGURE 3:**
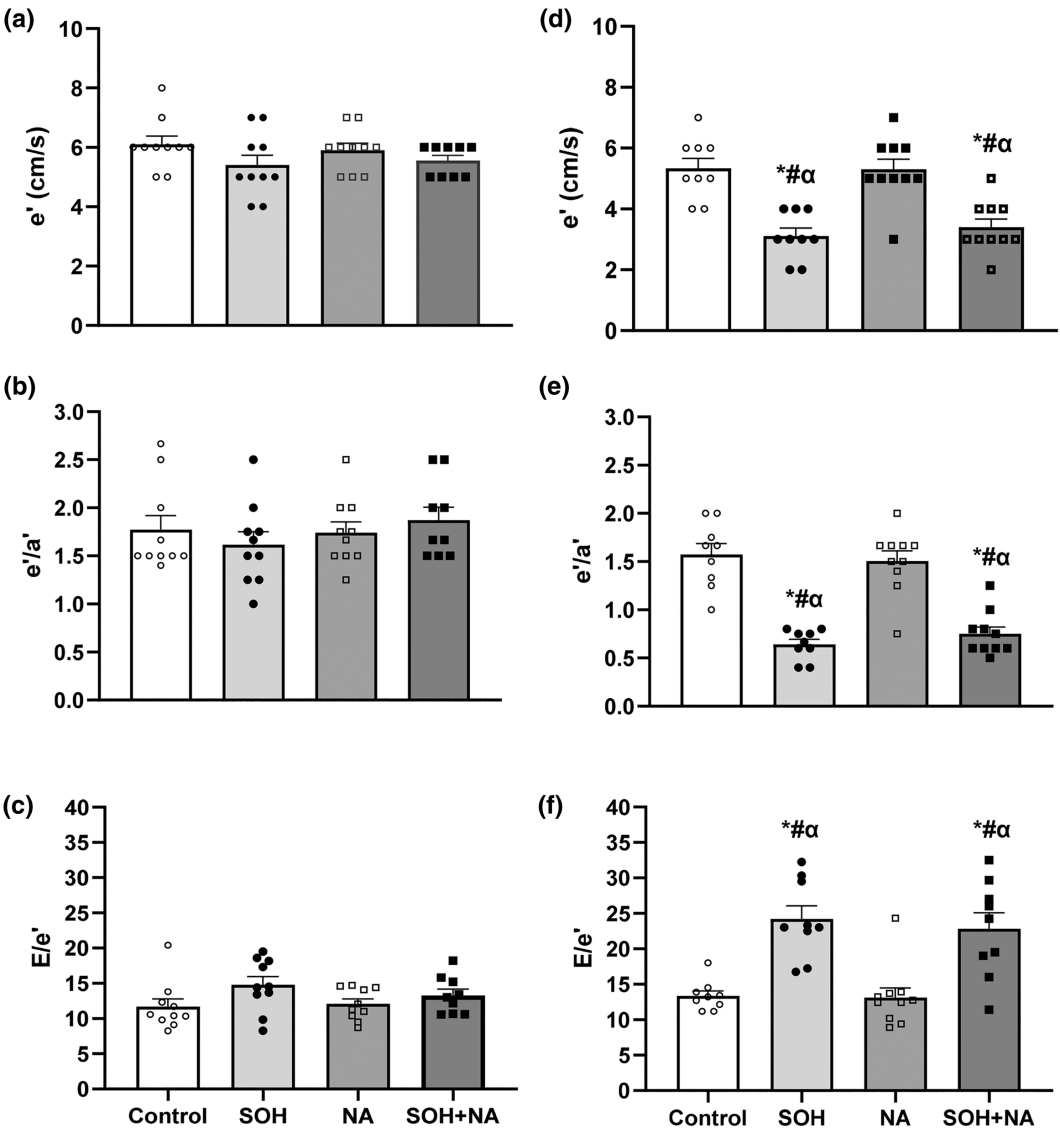
Effects of sweetened alcohol and naringin on lateral e' (a and d), e'/a' (b and e), and E/e' (c and f) in male (left panel) and female (right panel) Sprague–Dawley rats. Data presented as mean ± SEM. *p < 0.0001 compared to control. ^#^ *p* < 0.0001 compared to NA. ^α^ *p* < 0.0001 compared to all male groups. LV, left ventricular; NA, naringin; SOH, sweetened alcohol; SOH + NA, sweetened alcohol and naringin. e', peak velocity during early diastole; e'/a'; ratio of early to late peak velocities during diastole; E/e', ratio of early diastolic filling to peak velocity during early diastole.

Figure 3 shows the effects of sweetened alcohol and naringin on tissue Doppler indices in male and female Sprague–Dawley rats. The group and sex effects were significantly different for lateral e' (group: *F* = 15.41, *p* < 0.0001; sex: *F* = 52.18, *p* < 0.0001), e'/a' (group: *F* = 16.94, *p* < 0.0001; sex: *F* = 61.35, *p* < 0.0001), and E/e' (group: *F* = 14.25, *p* < 0.0001; sex: *F* = 34.55, *p* < 0.0001) in male and female rats.

There were no significant differences in all tissue Doppler indices in male rats (Figure 3a–c). Female SOH (mean ± SEM: 3.11 ± 0.29 cm/s) and SOH + NA (mean ± SEM: 3.40 ± 0.28 cm/s) groups had reduced lateral e' compared to control (mean ± SEM: 5.33 ± 0.29 cm/s) and NA (mean ± SEM: 5.30 ± 0.28 cm/s) groups (*p* < 0.0001; Figure 3d). Female SOH (mean ± SEM: 0.64 ± 0.12) and SOH + NA (mean ± SEM: 0.75 ± 0.11) groups had reduced e'/a' compared to control (mean ± SEM: 1.57 ± 0.12) and NA (mean ± SEM: 1.51 ± 0.11) groups (All *p* < 0.0001; Figure 3e). Female SOH (mean ± SEM: 24.21 ± 1.37) and SOH + NA (mean ± SEM: 23.16 ± 1.31) groups had increased E/e' compared to control (mean ± SEM: 13.35 ± 1.37) and NA (mean ± SEM: 13.12 ± 1.31) groups (*p* < 0.0001; Figure 3f). No significant differences were observed between the female SOH and SOH + NA groups.

All male groups had higher e', e'/a' and reduced E/e' compared to female SOH and SOH + NA groups (All *p* < 0.0001).

### 3.5 Cardiomyocyte diameters

**FIGURE 4:**
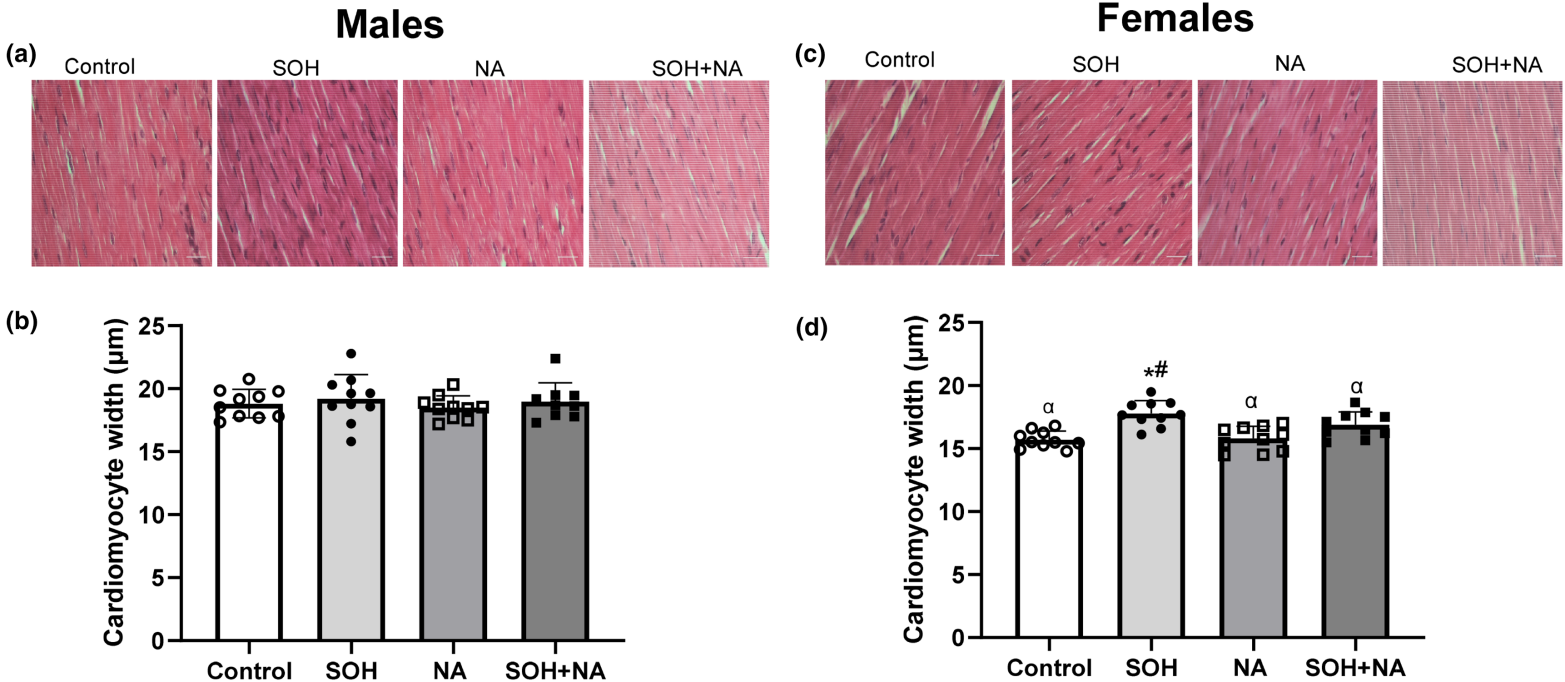
Effects of sweetened alcohol and naringin on LV cardiomyocyte diameters in longitudinal sections of male (left panel) and female (right panel) Sprague–Dawley rats. Representative H&E‐stained micrographs imaged at ×40 objective (×400 magnification) viewed in bright‐field (a and c). LV cardiomyocyte diameters (b and d) measured from the H&E‐stained sections. Data presented as means ± SEM. *p < 0.05 compared to control. ^#^ *p* < 0.05 compared to NA. ^α^ *p* < 0.0001 compared to all male groups. LV, left ventricle; NA, naringin; SOH + NA, sweetened alcohol and naringin; SOH, sweetened alcohol.

Figure 4 shows the effects of sweetened alcohol and naringin on LV cardiomyocyte diameters in longitudinal sections in male and female Spraque‐Dawley rats. The group and sex effects were significantly different for the LV cardiomyocyte diameters (group: *F* = 14.13, *p* < 0.0001; sex: *F* = 75.39, *p* < 0.0001) in male and female rats. There were no statistical differences in LV cardiomyocyte diameters between the groups in male rats (Figure 4a,b). In females, LV cardiomyocyte diameters were significantly greater in the SOH (mean ± SEM: 17.79 ± 0.38 μm) compared to the control (mean ± SEM: 15.71 ± 0.38 μm; *p* = 0.005) and NA groups (mean ± SEM: 15.81 ± 0.38 μm; *p* = 0.008; Figure 4c,d). All male groups had greater LV cardiomyocyte diameters compared to the female control, NA, and SOH + NA groups (*p* < 0.05).

### 3.6 Collagen area fraction

**FIGURE 5:**
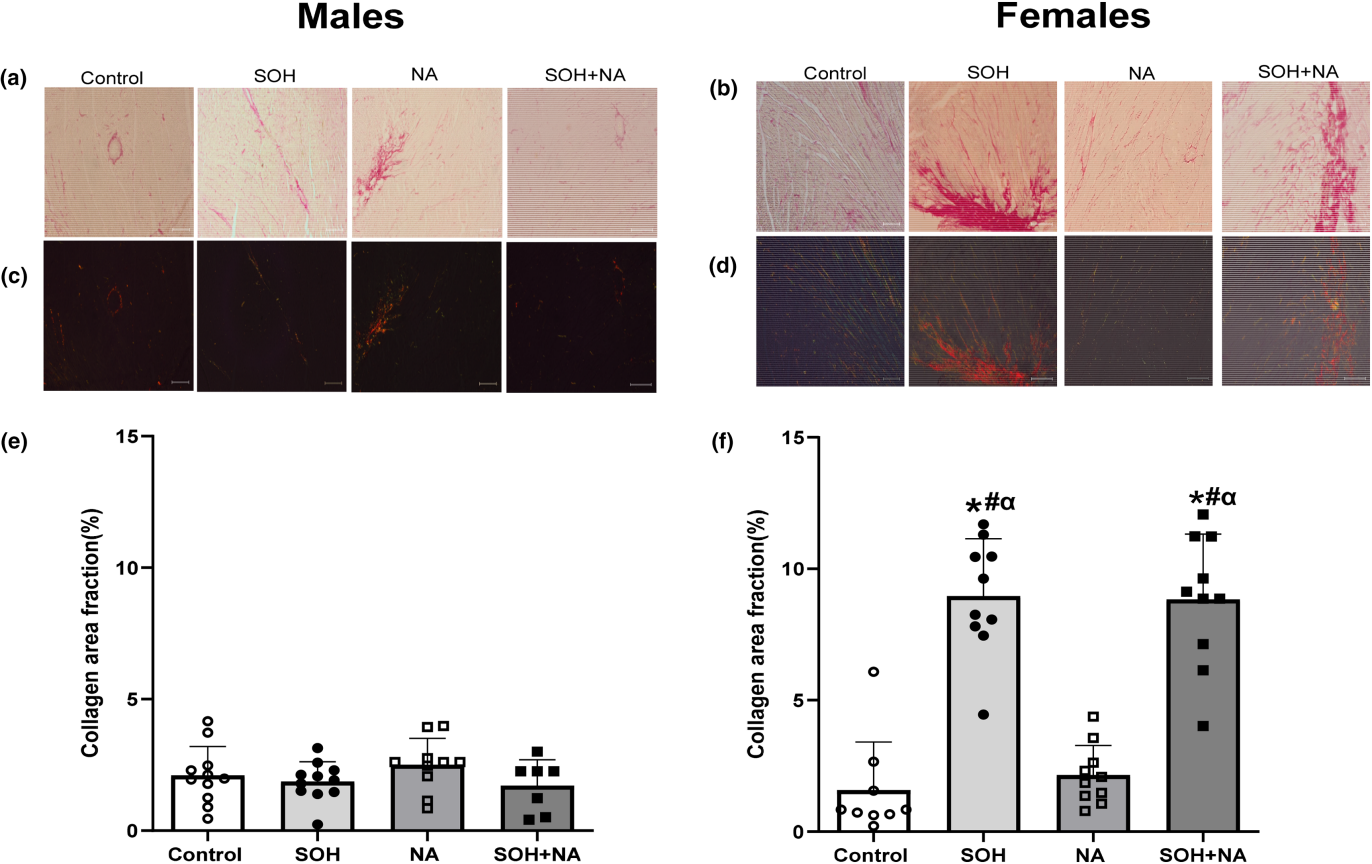
Effects of sweetened alcohol and naringin on collagen area fraction in LV tissue of male and female Sprague–Dawley rats. Representative picrosirius red stained micrographs imaged at ×10 objective (×100 magnification) viewed in bright‐field (a and b) and under polarized light (c and d). Collagen area fraction (e and f) calculated from picrosirius red‐stained sections. Data presented as means ± SEM. *p < 0.05 compared to control. ^#^ *p* < 0.05 compared to NA. ^α^p < 0.05 compared to all male groups. LV, left ventricle; NA, naringin; SOH + NA, sweetened alcohol and naringin; SOH, sweetened alcohol.

Figure 5 shows the effects of sweetened alcohol and naringin on LV collagen area fraction in male and female Sprague–Dawley rats. There were no visual differences in collagen accumulation between the groups of male rats when observed under bright filled (Figure 5a) or polarized light (Figure 5c). There was greater collagen accumulation in female SOH and SOH + NA rats compared to control and NA rats as evidenced by the increased red staining of cardiac tissue section visualized under bright‐filled microscopy (Figure 5b) and large collagen fibers in orange or yellow visualized under polarized light (Figure 5d).

The group and sex effects were significantly different for the collagen area fraction (group: *F* = 40.53, *p* < 0.0001; sex: *F* = 87.89, *p* < 0.0001) in male and female rats. There were no significant differences in the collagen area fraction between the groups in male rats (Figure 5e). In females, the collagen area fraction was significantly greater in the SOH group (mean ± SEM: 9.12 ± 0.52%) compared to the control (mean ± SEM: 1.58 ± 0.52%; *p* < 0.0001), and NA (mean ± SEM: 2.15 ± 0.51%; *p* < 0.0001) groups (Figure 5f). In females, the collagen area fraction was significantly greater in the SOH + NA group (mean ± SEM: 8.71 ± 0.47%) compared to the control (mean ± SEM: 1.58 ± 0.52%; *p* < 0.0001) and NA (mean ± SEM: 2.15 ± 0.51%; *p* < 0.0001) groups (Figure 5f). No significant differences in collagen area fraction were observed between SOH and SOH + NA female groups (*p* = 0.99; Figure 5f).

All male groups had reduced collagen area fraction compared to female SOH and SOH + NA groups (All *p* < 0.0001).

### 3.7 Associations between the collagen area fraction and tissue Doppler indices

**FIGURE 6:**
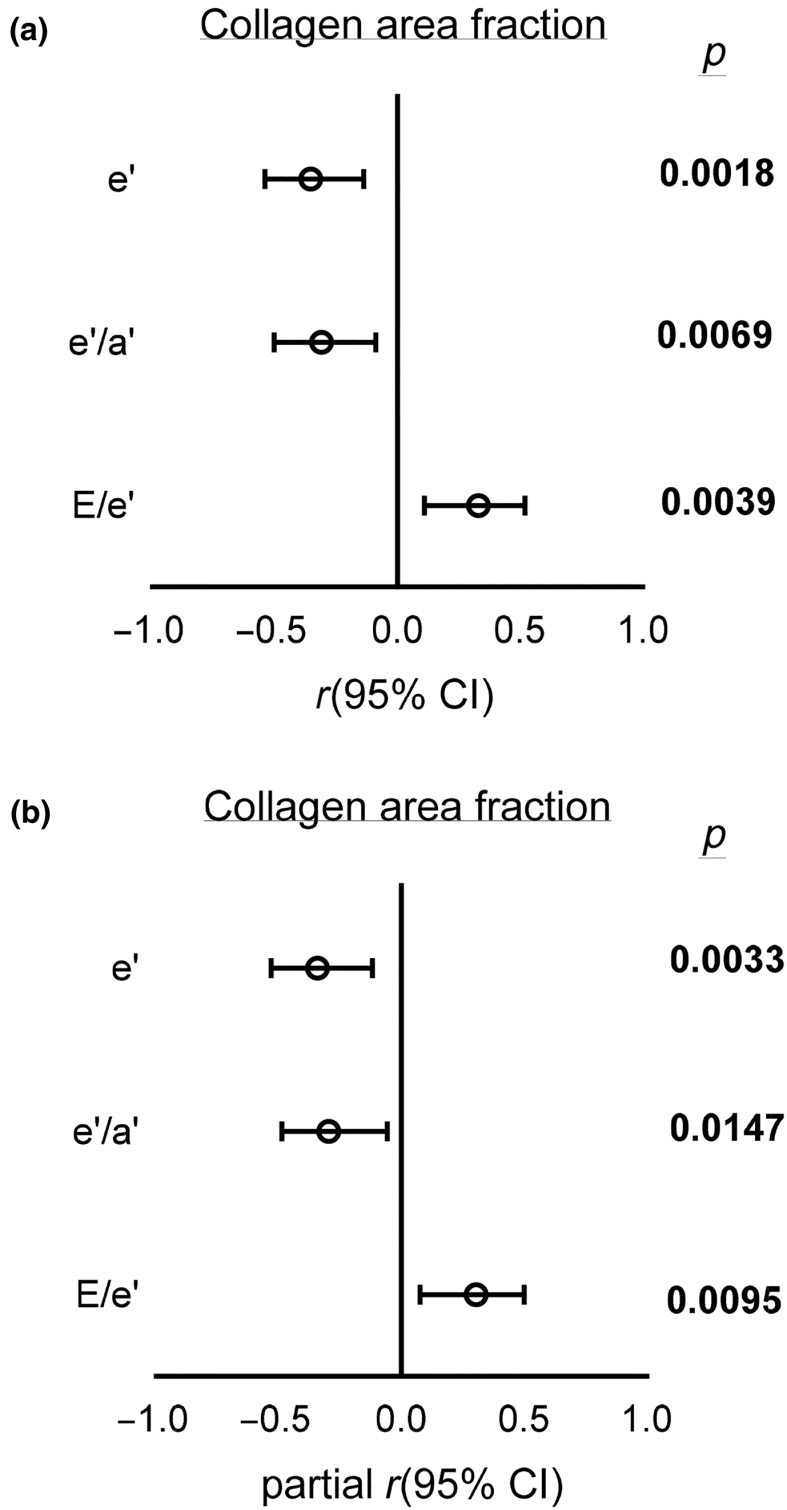
Associations between the collagen area fraction and measures of left ventricular diastolic function. (a) Before adjusting for BP, (b) After adjusting for BP. e', peak velocity during early diastole; e'/a'; ratio of early to late peak velocities during diastole; E/e', ratio of early diastolic filling to peak velocity during early diastole. Open circles represent the correlation coefficient (*r* or partial *r*), and horizontal lines represent the 95% confidence intervals (CI).

Figure 6 shows the associations between the collagen area fraction and tissue Doppler indices. Reduced e' was associated with increased collagen area fraction (*r* = −0.35; *p* = 0.0018). Reduced e'/a' was associated with collagen area fraction (*r* = −0.31; *p* = 0.0069). Increased E/e' was associated with collagen area fraction (*r* = 0.33; *p* = 0.0039; Figure 6a). After adjusting for blood pressure, the results remained materially unaltered (e': partial *r* = −0.34 and *p* = 0.0033; e'/a': partial *r* = −0.30 and *p* = 0.0147; E/e': partial *r* = 0.31 and *p* = 0.0095; Figure 6b).

## 4 DISCUSSION

The current study investigated the effects of sweetened alcohol and naringin on cardiac function in male and female adolescent Sprague–Dawley rats. The main findings of this study were that sweetened alcohol and naringin did not affect gelatine consumption in male rats. However, in female rats, the combination of sweetened alcohol and naringin led to decreased gelatine consumption. In males, sweetened alcohol or naringin did not exert an impact on cardiac geometry, systolic function, or diastolic function. However, in females, the consumption of sweetened alcohol led to concentric remodeling without inducing alterations in LV mass and blood pressure. Although sweetened alcohol did not affect systolic function, it impaired LV relaxation and led to elevated LV filling pressures in females. Increased collagen area fraction was associated with impaired LV relaxation and elevated filling pressures. Naringin may have the potential to improve the sweetened alcohol‐induced concentric remodeling, but not diastolic dysfunction in females. These findings highlight potential sex‐specific differences in cardiac structure and function in response to exposure to sweetened alcohol and naringin.

### 4.1 Effects of sweetened alcohol and naringin on gelatine consumption

Relative gelatine consumption, which accounts for body weight, was not significantly different among the male groups. This indicates that when adjusted for body mass, the male rats consumed gelatine in similar proportions regardless of their experimental condition. This uniformity suggests that the interventions did not markedly affect the dietary behavior of male rats in terms of gelatine consumption. In contrast, significant differences were noted in the relative gelatine consumption among female rats. Specifically, females in the SOH + NA group had reduced relative gelatine consumption compared to both the control and NA groups in week 8 and 9 of the study, however, was similar in the 10th week. It is unclear why this transient reduction in gelatine consumption in the SOH + NA group occurred. It does suggest that the combination of SOH and NA might influence the feeding behavior in female rats. Indeed, differences in preference for alcohol and fructose have previously been reported in rats (Li et al., 2019; Nieto & Kosten, 2017).

### 4.2 Effects of sweetened alcohol on cardiac geometry

In the current study, no differences were observed in markers of cardiac geometry in adolescent male rats. However, in adolescent females, the consumption of sweetened alcohol increased LV end‐diastolic posterior wall thickness and relative wall thickness compared to controls without altering heart and LV masses. In addition, the consumption of sweetened alcohol marginally increased the LV cardiomyocyte diameters. These results suggest that the consumption of sweetened alcohol may have caused concentric remodeling in females. Indeed, in concentric remodeling, LV wall thickness and relative wall thickness increase before any detectable changes in LV mass. The individual effects of a high‐fructose diet and alcohol on cardiac remodeling are controversial. While some studies have reported eccentric cardiac remodeling characterized by reduced LV wall thickness and relative wall thickness in rats subjected to fructose (Bouchard‐Thomassin et al., 2011; Komnenov et al., 2020) or alcohol (Ai et al., 2020; Kim et al., 2003; Mouton et al., 2020), other studies have reported concentric cardiac remodeling and hypertrophy in rats exposed to fructose (Wang et al., 2023; Xing et al., 2010) or alcohol (Fernández‐Solà & Porta, 2016). Interestingly, Wu et al. (2018) reported that the coadministration of fructose and alcohol (6% fructose +4% ethanol) led to improvements in myocardial hypertrophy compared to rats only consuming a high fructose diet (6% fructose) (Wu et al., 2018). The discrepancies in these findings may be due to specific variations in dietary conditions, age, as well as differences in rat strain and sex. Nevertheless, in the current study, we found that long‐term consumption of sweetened alcohol may result in more pronounced LV mass changes and concentric hypertrophy in females.

Although hypertension is a significant contributor to the cardiac remodeling process (Tomek & Bub, 2017), in the present study, blood pressures were similar between the control and SOH groups. This suggests that concentric remodeling in females occurred independent of changes in blood pressure. Indeed, cardiac remodeling can be induced not only by hypertension but also by inflammatory and fibrotic responses (Wang et al., 2023). In the current study, the consumption of sweetened alcohol increased LV total collagen content compared to the control group. Indeed, high fructose (Wang et al., 2023; Xing et al., 2010) or alcohol consumption (Lluís et al., 2011; Mouton et al., 2020) have been associated with cardiac fibrosis. In the heart, both fructose and alcohol may induce oxidative stress and inflammation, which then activate pathways that promote increased collagen formation and fibrosis (Kang et al., 2015; Zhang et al., 2016). Therefore, our findings suggest that the consumption of sweetened alcohol resulted in increased myocardial fibrosis which could have led to concentric remodeling in females.

### 4.3 Effects of sweetened alcohol on diastolic function

In the current study, sweetened alcohol did not affect pulsed Doppler indices in male and female Sprague–Dawley rats. In agreement with our study, Wu et al. (2018) reported no changes in the E/A ratio in 8‐week‐old rats exposed to sweetened alcohol (6% fructose +4% ethanol) for 8 weeks (Wu et al., 2018). Despite no changes in pulsed Doppler indices, the consumption of sweetened alcohol in the current study led to impaired tissue Doppler indices including reduced lateral e' and e'/a' and increased E/e' in female rats. In addition, increased collagen area fraction was associated with impaired LV relaxation and elevated filling pressures.

Although Wu et al. (2018) did not report on tissue Doppler indices, previous studies have reported impaired LV relaxation (reduced lateral e') and increased filling pressures (E/e') in rats fed a high fructose diet (Xing et al., 2010) and alcohol (Catena et al., 2016). In this regard, the consumption of sweetened alcohol may have impaired passive LV relaxation by promoting excessive collagen production and deposition that in turn, increased myocardial fibrosis. The increased myocardial fibrosis and impaired LV relaxation and stiffness caused by the sweetened alcohol may therefore lead to increased LV filling pressures (E/e'). In the current study, sweetened alcohol increased LV pressures despite the normal appearance of the E/A ratio. Indeed, increases in LV filling pressures drive a compensatory increase in the early diastolic filling (E), resulting in a pseudonormal filling pattern. Therefore, in the present study, the consumption of sweetened alcohol most likely caused moderate‐to‐severe diastolic dysfunction.

The mechanisms by which fructose and alcohol impair LV relaxation and increase filling pressures are multifactorial. In addition to promoting fibrosis‐mediated myocardial stiffness, fructose and alcohol may contribute to diastolic dysfunction by modifying calcium homeostasis, impacting contractile proteins, inducing oxidative stress, increasing lipogenesis, and promoting lipid accumulation in the myocardium (Kang et al., 2015; Laonigro et al., 2009; Li et al., 2022; Rasoul et al., 2023; Tsermpini et al., 2022; Wang et al., 2015, 2023; Zhang et al., 2016). Future studies should investigate these molecular mechanisms in more detail to gain a deeper understanding of their specific contributions.

### 4.4 Effects of sweetened alcohol on systolic function

In this study, the consumption of sweetened alcohol and naringin did not affect systolic function in both male and female rats. While some studies have reported that the individual consumption of fructose (Wang et al., 2023; Zhang et al., 2016) and alcohol (Ai et al., 2020; Kim et al., 2003; Mouton et al., 2020; van Oort et al., 2020) can adversely affect systolic function, characterized by impaired stroke volume, ejection fraction, and fractional shortening, others have not (Wang et al., 2023; Zhang et al., 2016). The majority of studies that have reported reduced ejection fraction and fractional shortening (Ai et al., 2020; Li et al., 2016; Wang et al., 2023; Zhang et al., 2016) have administered fructose and alcohol at high doses, and for a longer duration of intervention, which could partly explain the discrepancies in the results.

Nonetheless, similar to our results, Wu et al. (2018) reported no changes in systolic function in rats receiving sweetened alcohol. The assessment of systolic function in clinical practice often relies on LV ejection fraction, a measure influenced by chamber size and pressure. As a result, it serves as a more reliable indicator for evaluating ventricular‐arterial coupling than contractility (Borlaug et al., 2009). In our study, the use of more sensitive echocardiographic parameters, specifically Speckle Tracking Imaging (Dandel et al., 2009), may have facilitated the early detection of LV systolic dysfunction. In this context, research has indicated that myocardial deformation properties, specifically LV circumferential and longitudinal strain and strain rate may be compromised before any noticeable changes in systolic chamber function (Kucuk et al., 2017). Future studies should therefore use more sensitive measures of systolic function to explore the effect of sweetened alcohol on systolic performance.

### 4.5 Sex‐specific effects of sweetened alcohol on cardiac remodeling and diastolic function

Estrogen possesses anti‐inflammatory and vasodilatory properties, producing a cardioprotective environment for women (Iorga et al., 2017; Xiang et al., 2021). Nevertheless, our study showed that sweetened alcohol caused concentric remodeling and diastolic dysfunction in female rats, and not in their male counterparts. One plausible explanation for this discrepancy may be due to the interplay between sweetened alcohol and other physiological factors, specifically visceral adiposity. In the current study, female SOH rats had increased visceral adiposity indexed to body mass compared to male rats. Indeed, studies have reported that fructose consumption has a more pronounced effect on visceral adiposity in female rats compared to males (Kovačević et al., 2021). In addition, the accumulation of excess visceral adipose tissue plays an important role in the development of diastolic dysfunction and heart failure with a preserved ejection fraction in women more than in men (Sorimachi et al., 2021).

Hypertrophic adipocytes, prevalent in visceral adiposity may secrete inflammatory markers such as tumor necrosis factor‐alpha, interleukin 1β, interleukin 6, adipokines such as adiponectin, leptin, resistin, chemotactic protein 1 (MCP‐1), and transforming growth factor (TGF)‐β, (Farkhondeh et al., 2020) thereby promoting a state of chronic low‐grade inflammation (Kolb, 2022). The increased circulating levels of these inflammatory markers may cause damage to the endothelium leading to the upregulation of soluble intercellular adhesion molecules that increase the production of ROS (Paulus & Tschöpe, 2013). ROS decreases the bioavailability of nitric oxide and increases the production of peroxynitrites (Schulz et al., 2008). This in turn reduces the production of cyclic guanosine monophosphate and lowers protein kinase G (PKG) activity within the myocardium (van Heerebeek et al., 2012). These altered signaling pathways and PKG activity result in increased cardiac remodeling, diastolic stiffness, and dysfunction through downstream hypophosphorylation of the protein, titin (Paulus & Tschöpe, 2013). This ultimately leads to impaired diastolic function and concentric cardiac remodeling. Therefore, the sweetened alcohol‐induced increased visceral adiposity may have triggered a cascade of inflammatory events leading to the remodeling process in females, thus, increasing their susceptibility to diastolic dysfunction.

These results shed light on the complexity of gender‐specific responses to sweetened alcohol‐induced cardiac effects and highlight the importance of gaining a better understanding of the underlying physiological mechanisms contributing to diastolic function impairment and cardiac remodeling in female rats. Further studies are crucial to elucidate the interactions between visceral adiposity, inflammation, and other contributing factors that collectively influence the observed gender‐specific outcomes in response to sweetened alcohol administration.

### 4.6 Effects of naringin on sweetened alcohol‐induced concentric remodeling and diastolic dysfunction

In the current study, naringin may have potentially improved the sweetened alcohol‐induced concentric remodeling as the LV posterior wall thickness, relative wall thickness, and LV cardiomyocyte diameters in the SOH + NA group was similar to that of the controls. Previous studies have shown the potential therapeutic and cardioprotective effects of naringin in various experimental models of diet‐induced cardiac injury, diabetic cardiomyopathy, and ischemic heart diseases (Malakul et al., 2018; Park et al., 2018; Viswanatha et al., 2022).

Park et al. (2018) reported that naringin prevented the generation of ROS and mitochondrial dysfunction in cardiomyocytes exposed to fructose, which in turn decreased the hypertrophy of the cardiomyocytes (Park et al., 2018). Moreover, naringin prevented fructose‐induced cardiomyocyte apoptosis by obstructing ROS‐dependent ATM‐mediated p53 signaling (Park et al., 2018). To the best of our knowledge, the cardioprotective effects of naringin have not been explored in alcoholic models.

Although naringin may have marginally improved the sweetened alcohol‐induced concentric remodeling, it did not improve the severity of fibrosis since the total collagen content was similar in SOH + NA and SOH groups but were both significantly higher compared to controls. Therefore, it is plausible that naringin did not directly impact collagen formation, but rather it may have improved indices of concentric remodeling through alternative mechanisms, such as the reduction in ROS formation as reported in other studies (Akin et al., 2022; Bruno et al., 2022; Laonigro et al., 2009). Future studies should consider these mechanisms to further explore the potential of naringin in the improvement of sweetened alcohol‐induced concentric remodeling at a molecular level.

Although naringin may have marginally improved concentric remodeling, it did not ameliorate the sweetened alcohol‐induced diastolic dysfunction. Concentric remodeling has the potential to contribute to diastolic dysfunction, as the increased stiffness of the heart muscle may affect its ability to relax during diastole (Røe et al., 2017). Given the observed improvement in sweetened alcohol‐induced concentric remodeling with naringin, a longer duration of treatment and higher doses of naringin may have eventually led to improvements in diastolic function. Therefore, these factors should be considered in future studies. Additionally, understanding the broader metabolic responses that lead to reduced gelatine consumption in females could provide insights into the interconnected nature of diet and cardiac health in the presence of such substances.

The present study has further limitations. While previous studies have extensively examined the individual effects of fructose (Bouchard‐Thomassin et al., 2011; Kang et al., 2015; Komnenov et al., 2020; Wang et al., 2023; Xing et al., 2010; Zhang et al., 2016) and alcohol (Ai et al., 2020; Fernández‐Solà & Porta, 2016; Kim et al., 2003; Mouton et al., 2020; Nieto & Kosten, 2017) on cardiac function, these studies did not employ gelatine as a vehicle. Future investigations would benefit from the incorporation of 20% fructose and 10% ethanol solely within gelatine to provide a clearer comparison between individual and combined effects. In addition, the use of a single dose or concentration for each compound of interest (fructose at 20%, alcohol at 10%, and naringin at 50 mg/kg) may have contributed to the absence of some of the observable effects. Future investigations need to explore a wider range of doses to elucidate potential dose–response relationships more comprehensively. As Gomori's trichrome and wheat germ agglutinin (WGA) stains offer better specificity and quantitative accuracy for measuring cardiomyocyte size compared to the more general H&E staining, further investigation in this regard may provide additional insights in the assessment of cardiac remodeling. Finally, the lack of measurement of blood alcohol concentration following consumption of ethanol‐containing gelatine is an aspect that warrants further investigation in future studies since insights into how gelatine matrices influence alcohol release and absorption can inform alcohol metabolism considerations and health implications.

## 5 CONCLUSION

In males, neither sweetened alcohol nor naringin had any impact on cardiac geometry or diastolic function. Conversely, in females, the consumption of sweetened alcohol resulted in increased visceral obesity, and concentric remodeling without inducing alterations in LV mass and blood pressure. Sweetened alcohol‐impaired LV relaxation and elevated LV filling pressures in females. Notably, naringin improved the sweetened alcohol‐induced concentric remodeling but did not mitigate the diastolic dysfunction and visceral obesity induced by sweetened alcohol in females. These findings contribute to a better understanding of the effects of sweetened alcohol and naringin on cardiac health and underscore the need for further research to unravel the underlying mechanisms that account for gender‐specific differences.

## AUTHOR CONTRIBUTIONS

JM collected and analyzed the data and drafted the manuscript. KHE, KGI designed the research, collected the data, and reviewed the manuscript. LM designed the research, collected, and analyzed the data, and reviewed the manuscript.

## FUNDING INFORMATION

This work was supported by the University of the Witwatersrand internal funding sources (Faculty of Health Sciences Research Committee of the University of Witwatersrand [grant number: 001.401.8521101.0000000.000000 PHSMFRO], The School of Physiology University of the Witwatersrand Research Incentive funds [grant number: 001.169.8521101.5121105.000000.0000000000.4463]), the National Research Foundation (NRF) of South Africa Thuthuka Fund (grant number: TTK200323510481), The Federal University Birnin Kebbi (Nigeria) and the PhD funding for the candidate by the Tertiary Education Trust Fund of Nigeria.

## CONFLICT OF INTEREST STATEMENT

The authors declare that they have no competing interests.

## ETHICS STATEMENT

The study adhered to international ethical guidelines and standards governing the care and utilization of laboratory animals, receiving approval from the Animal Research Ethics Committee of the University of Witwatersrand (AREC clearance certificate number 2022/10/02/C). The research took place at the Wits Research Animal Facility situated at the University of the Witwatersrand in Johannesburg, South Africa.
